# Brugada Syndrome in a Patient with Vascular Ehlers-Danlos Syndrome: Sudden Death Risk Amplified

**DOI:** 10.7759/cureus.1178

**Published:** 2017-04-19

**Authors:** Jason D'Souza, Divyanshu Malhotra, Aditya Goud, Chanukya Dahagam, George Everett

**Affiliations:** 1 Internal Medicine, Florida Hospital-Orlando; 2 Nephrology, Yale New Haven Hospital; 3 Internal Medicine, Medstar Franklin Square Medical Center, Baltimore, MD

**Keywords:** brugada syndrome, ehler-danlos syndrome, sudden death

## Abstract

The vast majority of sudden cardiac arrests occur in patients with structural heart disease and in approximately 10% of the cases, it can occur in those with structurally normal hearts. Brugada syndrome is an autosomal dominant sodium channelopathy that has been implicated in sudden deaths. Given their low prevalence, our knowledge about Brugada syndrome is still evolving. Apart from schizophrenia, there have been no reports of associated medical conditions. We recently encountered a patient with vascular Ehlers-Danlos syndrome who was also found to have Brugada syndrome. Both these conditions share some common clinical presentations including a propensity for sudden death.

## Introduction

Brugada syndrome is a rare autosomal dominant sodium channelopathy of apparently structurally normal hearts [1]. The true prevalence of Brugada syndrome is not known. Estimates of prevalence are uncertain because variable electrocardiogram (EKG) changes can be seen in 40% of the patients. The prevalence ranges from 0.14% in the Japanese population to 0.61% in Europeans. The worldwide prevalence of this EKG pattern in the general population has been estimated to be one in every 1000 individuals [2-3]. However, the coexistence of this disorder with other medical conditions has not been demonstrated with the exception of schizophrenia which was noted in a cross-sectional study by Blom, et al. [4]. To the best of our knowledge, we are reporting the index case of Brugada syndrome coexisting with another rare autosomal dominant genetic condition, vascular Ehlers-Danlos syndrome. Informed consent statement was obtained for this study.

## Case presentation

**Figure 1:**
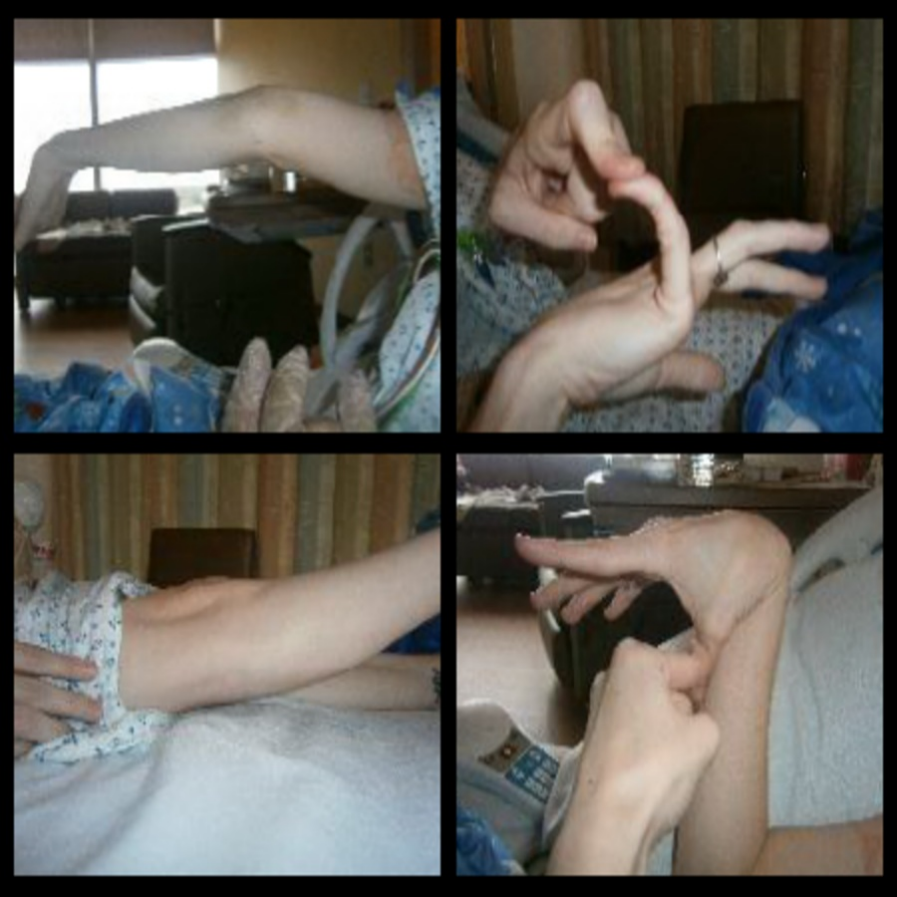
Examination for joint hypermobility In clockwise direction: hyperextension of the elbow > 10°, dorsiflexion of the fifth finger > 90°, passive apposition of the thumb to the flexor aspect of the forearm, and hyperextension of the knee > 180°

**Figure 2:**
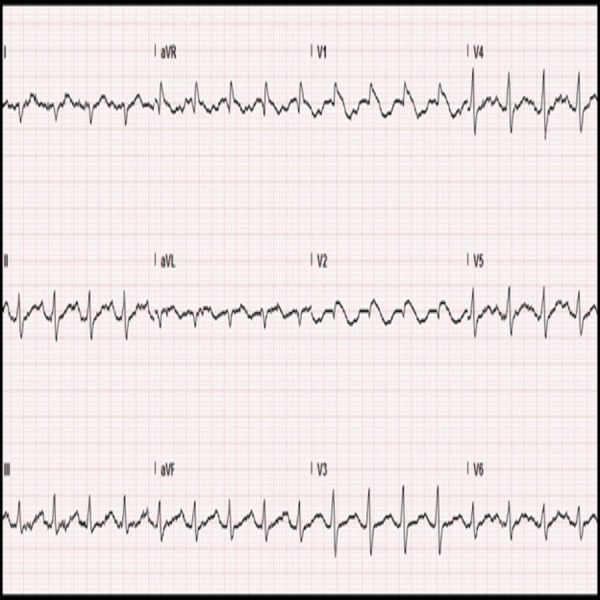
Electrocardiogram (EKG) EKG of the patient while she was experiencing chest pain. Note the pseudo-right bundle branch block pattern in V1 with a coved type ST elevation in V1, V2 and associated T-wave inversions in these two leads

A 22-year-old Caucasian female with a past history of multiple syncopal episodes presented to the emergency department with chest pressure and palpitations. She did not have any other accompanying symptoms. One week previously, she had an episode of self-limited fever and coryza. Her past medical history included a repaired congenital clubfoot and Ehlers-Danlos syndrome – vascular subtype (diagnosis confirmed by genetics). Her previous syncopal episodes had been attributed to atrial arrhythmias, postural orthostatic tachycardia syndrome (POTS) or to neuro cardiogenic syncope. Flecainide and fludrocortisone had been previously prescribed to her for atrial arrhythmias and POTS respectively. Family history included the sudden cardiac death of her maternal grandfather and great-grandfather, both prior to the age of 50. Physical examination was remarkable for thin translucent skin and a thin pinched-up nose. She was able to dorsiflex her fifth finger to more than 90°, could perform passive opposition of her thumb to the flexor aspect of the forearm, was able to hyperextend her elbow greater than 10° and was also able to hyperextend the knee greater than 180° (Figure 1). She thus secured eight out of nine points for the Beighton hypermobility score (Table 1). Her basic diagnostic studies, except for the EKG, were unremarkable. Her EKG at the time of presentation showed the classic Brugada type – one pattern as shown in (Figure 2). Her previous echocardiograms did not reveal any evidence of structural heart disease. Thus following the diagnosis of Brugada syndrome, flecainide was immediately discontinued and the patient was started on quinidine. She later underwent an implantable cardioverter defibrillator placement. Fourteen months into follow-up, the patient had multiple successfully aborted episodes of ventricular tachycardia and was doing well overall.

**Table 1:** Beighton score for joint hypermobility

Beighton Score for Joint Hypermobility	
Forward flexion of trunk, knees straight and palms touching the floor	1
Bilateral hyperextension of the knee > 10°	1 each
Bilateral hyperextension of the elbow > 10°	1 each
Bilateral passive apposition of the thumb to the flexor aspect of the forearm	1 each
Bilateral passive hyperextension of the of the fifth finger joint > 90°	1 each
Maximum possible score	9

## Discussion

Vascular Ehlers-Danlos syndrome is potentially life threatening and 80% of individuals with this condition experience a major vascular event by the age of 40 with a median age of death at 50. Currently, available statistics estimate the prevalence of this condition to be about one in 100,000 [5]. These statistics parallel those of Brugada syndrome which is also an important cause of sudden death. The average age at diagnosis of Brugada syndrome is about 41 years [6]. Both of these conditions are autosomal dominant with Brugada syndrome caused by mutations in cardiac sodium channel (SCN5A) and SCN10A encoding subunits of the cardiac sodium channel (gene locus 3p21-24) and Ehlers-Danlos vascular subtype being caused by heterozygous mutations in the collagen type III alpha 1 chain (COL3A1) gene (gene locus 2q31) encoding type III collagen [1,7]. Both these conditions are also associated with POTS [8]. In addition, they are also very strong considerations for sudden death cases in sports medicine [9-10]. Given that both these disorders share common properties and have potentially life-threatening implications, it could be that they have been under-represented in the statistics of sudden death when one cause has been implicated and the search for another has been abandoned. This is particularly plausible in scenarios where arrhythmias are a likely cause of death but cannot be confirmed retrospectively or in post-mortem studies. Given the currently reported prevalence of the two conditions, which may be underestimated due to the subtlety of clinical presentation, the likelihood that they would occur in a single individual is about one in 10^8^. 

## Conclusions

Both Brugada syndrome and vascular Ehlers-Danlos syndrome are potentially life-threatening conditions. The diagnosis of one rare cause of sudden death does not exclude the possibility of another co-existing life-threatening condition.
